# Peutz-Jegher’s syndrome presenting as jejunoileal intussusception in an adult male: a case report

**DOI:** 10.4076/1757-1626-2-8865

**Published:** 2009-08-11

**Authors:** Hardik H Thakker, Amita Joshi, Aparna Deshpande

**Affiliations:** 1 Seth G S Medical College and KEM hospitalParel, Mumbai 400012India; 2 Department of Pathology, Seth G S Medical College and KEM hospitalParel, Mumbai 400012India; 3 Department of General Surgery, Seth G S Medical College and KEM hospitalParel, Mumbai 400012India

## Abstract

**Introduction:**

Peutz-Jegher’s syndrome is a rare autosomal dominant disorder that typically manifests itself as recurrent colicky abdominal pain and blood loss in stools. In adults, it is only rarely accompanied by frank intussusception and intestinal obstruction. We encountered an adult Asian Indian male who presented with an intestinal obstruction due to jejunoileal intussusception. It was caused by a 3.5 cm large hamartomatous polyp of Peutz-Jegher’s syndrome. We feel reporting the unusual presentation of this rare condition may be a noteworthy contribution to the scarce literature on Peutz-Jegher’s syndrome from India. The case report may be of educational importance to the clinicians and students because it is unusual to see this case in typical clinical practice.

**Case presentation:**

A 38-year-old Asian Indian male presented to us in the surgical emergency room with colicky abdominal pain, reporting vomiting and blood in stools over the previous two days. Clinical examination suggested intestinal obstruction. Ultrasonography of the abdomen showed signs of intussusceptions, which were then confirmed by an emergency exploratory laparotomy. We resected the intussuscepted small bowel segment and performed a jejuno-ileal anastomosis. A histopathology examination of the resected specimen revealed multiple hamartomatous polyps suggestive of Peutz-Jegher’s syndrome. In this case report, we present the pathology findings, their clinical correlation and a detailed discussion of Peutz-Jegher’s syndrome and adult intussusception. We also discuss its other rare presentations reported in literature.

**Conclusion:**

Hamartomatous polyps of Peutz-Jegher’s syndrome can sometimes grow to a large size and form the lead point of an intussusception.

## Introduction

Peutz-Jegher’s syndrome is a rare autosomal dominant disorder with variable penetrance. It is characterized by mucocutaneous hyperpigmentation, hamartomatous polyps of the gastrointestinal tract and multiple neoplasms. It commonly presents as recurrent colicky abdominal pain and blood loss in stools. An adult Asian Indian patient presented to us with an intestinal obstruction. This was secondary to a large hamartomatous jejunal polyp causing intussusception. Only 5% to 15% [1,2,3] of intussusceptions occur in adults and they contribute to only 1% [4,5] of all causes of intestinal obstruction. A report of this unusual presentation of a rare condition may be a noteworthy contribution to the scarce literature on Peutz-Jegher’s syndrome from India.

## Case presentation

**Figure 1.**
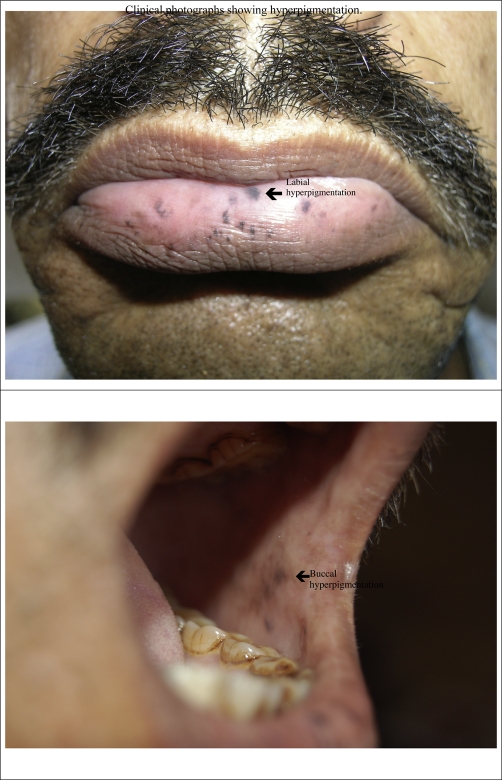
Shows clinical photographs of the patient with labial and buccal hyperpigmentation.

A 38-year-Asian Indian male presented to us in the emergency room with colicky abdominal pain in left lower and umbilical area, vomiting and blood in stools over the previous 2 days. He did not have a history of fever. He had a history of intermittent abdominal pain over the previous 2 months. His family history was not significant. On examination he was afebrile, with a pulse of 92/min regular and blood pressure of 130/88 mm Hg. The lower labial and buccal mucosa showed hyper pigmentation bilaterally (Figure 1). There was tenderness and guarding in the left iliac fossa. There was no lump, organomegaly or free fluid. Hernial orifices were normal. However, bowel sounds were increased.

Blood investigations showed severe anemia (Hb: 7 gm/dl). A plain x-ray of the abdomen was unremarkable. Abdominal ultrasonography showed two echo complex areas with concentric rings and target appearance: one in the left lumbar region and the other one of size 3.5 cm × 5 cm in the hypochondriac. This was suggestive of an intussusception.

We started the patient on intravenous fluids and antibiotics. We inserted a nasogastric tube and transfused him with two units of whole blood. We then performed an emergency exploratory laparotomy. Intra-operatively we found a one-foot-long segment of jejuno-ileal intussusception, extending approximately three feet distally from the duodenojejunal flexure. We resected the intussuscepted segment and performed a jejuno-ileal anastomosis. We found multiple polyps on the jejunal resection margins. We sent the resected segment for histopathology examination. We then placed a drain and closed the abdomen. His recovery in the wards was uneventful. We discharged him 8 days after the surgery.

### Gross pathology findings

**Figure 2.**
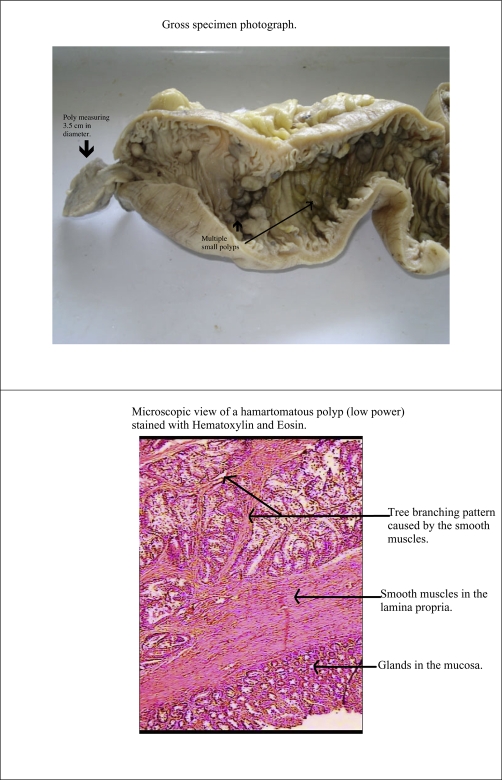
Shows a gross pathology photograph of the resected segment of the small bowel with a large hamartomatous polyp. This polyp formed the lead point for intussusception. It also shows a low power microscopic view of a hamartomatous polyp stained with Hematoxylin and Eosin.

The specimen of small intestine was 32 cm in length, with grey serosa and unremarkable cut section. There was no evidence of gangrenous changes. The mucosa was lined by multiple polyps that were scattered and on average measuring approximately 1 cm in diameter. A single large pedunculated polyp measuring 3.5 cm in diameter was attached to the mucosa with a stalk 1.1 cm in length, located 2 cm from the proximal resected margins. The polyps were reddish grey in color (Figure 2).

### Histopathology

Multiple sections studied from the polyps showed features of hamartomatous polyps, composed of glands separated by a band of smooth muscles (Figure 2). There was no evidence of atypia or malignancy. Lymph nodes showed reactive hyperplasia.

### Diagnosis

Peutz-Jegher’s Syndrome. We subjected him to regular follow up and a strict surveillance for other neoplasms common in Peutz-Jegher’s syndrome. We could not carry out a mutation analysis for STK 11/LKB 1 gene due to lack of resources.

## Discussion

The occurrence of adult intussusception is rare [1,2,3]. Its characteristics in adults are different from its characteristics in children in some notable respects as indicated in Table 1. Peutz-Jegher’s syndrome is a rare autosomal disorder with variable penetrance characterized by mucocutaneous hyper pigmentation, hamartomatous polyps of gastrointestinal tract and multiple neoplasms. The diagnostic criteria for the syndrome are as follows: a) Three or more histologically confirmed Peutz-Jegher’s polyps or b) Any number of Peutz-Jegher’s polyps with a family history or c) Characteristic prominent mucocutaneous pigmentation with a family history or d) Any number of Peutz-Jegher’s polyps with characteristic prominent mucocutaneous pigmentation. It commonly presents as recurrent colicky pain in abdomen and blood loss in stools. The polyps in this condition are hamartomatous and rarely undergo malignant transition but a few such cases have been reported in literature [6]. The patients are prone to many extra intestinal tumors like testicular sertoli tumors, ovarian tumors like sex cord tumors with annular tubules, granulose theca cell tumors, cystadenomas, breast tumors like breast carcinoma, papilloma with squamous metaplasia, cholangioma, pancreatic adenocarcinoma, adenoma malignum, bronchial carcinoids, papillomas in bladder and pelvis [7]. Patients observed with such conditions need to be placed under regular surveillance to facilitate early detection and regular followup.

**Table 1.** The characteristics of intussusception in adults and children

	Adult intussusception	Intussusception in children
Frequency	5% of all cases	95% of all cases
Part of the bowel involved	Commonly involves small bowel	Usually involved colon
Symptoms	Classical symptoms of intussusception are not always present	Classical symptoms are present in many cases
Cause	Caused by malignancy (50%) or polyp	Idiopathic due to lymphoid hyperplasia in Peyer's patches
Treatment	Treatment is usually resection of intussuscepted segment without reduction (to prevent seedling of malignant cells)	Treatment is hydrostatic reduction and if needed manual reduction by laparotomy, resection not needed unless gangrene sets in

Patients may also have other non neoplastic extra intestinal lesions like nasal polyps, gall bladder polyps, nodular goiter and adrenal cortical hyperplasia [7].

Recently, gene mutation studies have proved that mutation in Serine Threonine kinase gene STK 11/LKB 1 located on 19p.13.3 [8], is the cause of Peutz-Jegher’s. However, the exact mechanism by which it affects protein synthesis and causes polyp formation is yet to be elucidated. Cases of intussusception in Peutz-Jegher’s have been reported in children [9,10,11] but have been rare in adults. Cases of Peutz-Jegher’s with carcinoid [12], duodenal [13] and gastric [14] carcinomas have also been reported.

### Correlation of pathologic findings

The largest polyp measuring 3.5 cm in size was the lead point causing telescoping of jejunum into ileum, thereby causing abdominal pain, guarding and currant jelly stools. The anemia could be attributed to bleeding from the multiple hamartomatous polyps, but we could not rule out other causes like ulcer disease, and iron deficiency.

### Future advances

Once the exact mechanism by which mutations in STK 11/LKB1 cause Peutz-Jegher’s Syndrome is known, it will open doors of advance treatment modalities like gene therapy.

## Conclusion

Hamartomatous polyps of Peutz-Jegher’s syndrome can sometimes grow to a large size and form the lead point of an intussusception.

## Abbreviation

Hb: haemoglobin
